# Polygenic analysis of very high acetic acid tolerance in the yeast *Saccharomyces cerevisiae* reveals a complex genetic background and several new causative alleles

**DOI:** 10.1186/s13068-020-01761-5

**Published:** 2020-07-16

**Authors:** Marija Stojiljkovic, María R. Foulquié-Moreno, Johan M. Thevelein

**Affiliations:** 1 Laboratory of Molecular Cell Biology, Institute of Botany and Microbiology, Department of Biology, KU Leuven, Kasteelpark Arenberg 31, 3001 Leuven-Heverlee, Flanders Belgium; 2 Center for Microbiology, VIB, Kasteelpark Arenberg 31, 3001 Leuven-Heverlee, Flanders Belgium

**Keywords:** Bioethanol production, Acetic acid tolerance, Polygenic analysis, Industrial yeast

## Abstract

**Background:**

High acetic acid tolerance is of major importance in industrial yeast strains used for second-generation bioethanol production, because of the high acetic acid content of lignocellulose hydrolysates. It is also important in first-generation starch hydrolysates and in sourdoughs containing significant acetic acid levels. We have previously identified *snf4*^E269*^ as a causative allele in strain MS164 obtained after whole-genome (WG) transformation and selection for improved acetic acid tolerance.

**Results:**

We have now performed polygenic analysis with the same WG transformant MS164 to identify novel causative alleles interacting with *snf4*^E269*^ to further enhance acetic acid tolerance, from a range of 0.8–1.2% acetic acid at pH 4.7, to previously unmatched levels for *Saccharomyces cerevisiae*. For that purpose, we crossed the WG transformant with strain 16D, a previously identified strain displaying very high acetic acid tolerance. Quantitative trait locus (QTL) mapping with pooled-segregant whole-genome sequence analysis identified four major and two minor QTLs. In addition to confirmation of *snf4*^E269*^ in QTL1, we identified six other genes linked to very high acetic acid tolerance, *TRT2*, *MET4, IRA2* and *RTG1* and a combination of *MSH2* and *HAL9*, some of which have never been connected previously to acetic acid tolerance. Several of these genes appear to be wild-type alleles that complement defective alleles present in the other parent strain.

**Conclusions:**

The presence of several novel causative genes highlights the distinct genetic basis and the strong genetic background dependency of very high acetic acid tolerance. Our results suggest that elimination of inferior mutant alleles might be equally important for reaching very high acetic acid tolerance as introduction of rare superior alleles. The superior alleles of *MET4* and *RTG1* might be useful for further improvement of acetic acid tolerance in specific industrial yeast strains.

## Background

Acetic acid is a major inhibitor in industrial yeast fermentations. Due to its high toxicity it is actually used as an antimicrobial preservative in the food industry [1]. Especially in lignocellulose hydrolysates, used in second-generation bioethanol production, acetic acid levels tend to be very high. Second-generation bioethanol production uses the lignocellulosic biomass derived from waste residues (bagasse, wheat and rice straw, paper pulp and other agricultural, forestry and industrial residues) or bioenergy crops as substrate. Lignocellulosic biomass consists mainly of cellulose, hemicellulose and lignin [2, 3]. The cellulosic polymers contain many acetyl groups that are released upon pretreatment and hydrolysis in the form of acetic acid. The levels of acetic acid in the hydrolysate vary depending on the lignocellulosic substrate used. In softwoods, acetyl groups are present in the *O*-acetyl-(galacto)glucomannans while in the hardwoods they are in the acetylated xylan. Moreover, softwoods generally have lower acetyl content than hardwoods and consequently generate lower levels of acetic acid in the hydrolysates. The pretreatment methodology used generates additional amounts of acetic acid as well as multiple other inhibitory chemicals. As a result, the overall concentration of acetic acid ranges in different lignocellulosic hydrolysates from 1 to 10 g/L [4–8]. In general, cheaper pretreatment methodologies generate higher amounts of inhibitors, including acetic acid. Furfural, HMF and acetic acid tend to be the main inhibitors in second-generation hydrolysates [3]. Also in first-generation bioethanol production with hydrolysates of starch derived from food crops or molasses, significant levels of acetic acid can be present. It is produced by contaminating bacteria and accumulates due to water recycling practices [9]. Tolerance to acetic acid is also important in baker’s yeast used in sourdoughs where acetic acid is produced in the preceding bacterial fermentation [10].

The pKA of acetic acid is 4.76. The protonated form present at low medium pH easily diffuses through membranes and dissociates in the cytosol with its neutral pH into a proton and an anion, causing a precipitous drop in intracellular pH [11, 12]. This causes wide-spread inhibition of many cellular functions, which can only be overcome by energy-dependent export of the protons and anions back into the medium [13–15]. In engineered industrial yeast strains used for bioethanol production with lignocellulosic hydrolysates, the artificial capacity of xylose fermentation turned out to be much more sensitive to acetic acid than fermentation of glucose [16–19]. Because of its importance as a fermentation inhibitor, many studies have focussed on the genetic elements underlying acetic acid tolerance and on experimental approaches to enhance acetic acid tolerance. The finding that acetic acid causes oxidative stress led to the discovery that pre-incubation with H_2_O_2_ [20] improved acetic acid tolerance, but the underlying mechanisms were not further explored. Similarly, genome shuffling [21], pre-adaptation [5] and evolutionary adaptation strategies [22, 23] have been used to enhance acetic acid tolerance without identification of the causative genetic changes. This also revealed the frequent instability of acetic acid-tolerant strains obtained by evolutionary adaptation [23]. Under appropriate conditions, however, evolutionary adaptation can also generate stable strains in which novel causative genes (*ASG1*, *ADH3*, *SKS1* and *GIS4*) were identified [24]. Early studies on acetic acid tolerance focused on proteins strongly induced by acetic acid, like the Pdr12 acetate exporter and the Pma1 plasma membrane H^+^-ATPase [25]. A major breakthrough was the identification of the Haa1 transcription factor that plays a crucial role in the acetic acid response and of which increased activity enhances acetic acid tolerance substantially [26–30]. Polygenic analysis of a yeast strain with very high acetic acid tolerance revealed *HAA1*, *VMA7*, *GLO1*, *DOT5* and *CUP2* as causative genes and suggested the existence of many other factors involved in high acetic acid tolerance [31]. This is also shown by the many reports on targeted improvement of acetic acid tolerance by modification of specific genes, such as point mutations in *HAA1* [29, 31], deletion/disruption of *JJJ1* [32] and *FPS1* [33]), and besides *HAA1,* overexpression of *WHI2* [34], *ACS2* [35], *RTC3* and *ANB1* [36]. A shortcoming of these studies is that they were mostly conducted with laboratory yeast strains or at most with first-generation bioethanol strains with relatively low acetic acid tolerance. This makes it unclear whether the reported genetic modifications would also be effective in industrial yeast strains with high inherent robustness against stress factors, like acetic acid, and in particular whether these modifications can further enhance the high acetic acid tolerance currently already achieved in industrial yeast strains used for second-generation bioethanol production.

Acetic acid tolerance is a quantitative polygenic trait defined by multiple genetic elements, of which some, like *HAA1*, can be directly linked to known mechanisms for acetic acid tolerance, but for many others the mechanism is unclear and their interaction is likely complex. A further complicating factor is the environmental dependency of acetic acid toxicity, with for instance the pH, temperature and presence of ethanol and other inhibitors as major interfering factors. Pooled-segregant whole-genome sequence analysis and reciprocal hemizygosity analysis (RHA) have been very effective in mapping quantitative trait loci (QTLs) and identifying their causative genetic elements for many complex traits in yeast [37, 38]. These include industrially relevant properties like thermotolerance [39–43], high ethanol tolerance [44], ethanol accumulation capacity [45], glycerol growth [46] and yield [47, 48], nitrogen consumption [49], flavor compound production [50–53], as well as acetic acid tolerance [31]. This has also been performed for acetic acid tolerance, with a whole range of genes affecting acetic acid tolerance now identified. However, these genes have been identified in different genetic backgrounds, with highly varying levels of intrinsic acetic acid tolerance and under widely different evaluation conditions. Hence, it has remained unclear to what extent acetic acid tolerance can be improved in *S. cerevisiae* under industrially relevant fermentation conditions, especially since some other yeast species, like the food spoilage yeast *Zygosaccharomyces bailii*, display much higher intrinsic acetic acid tolerance compared to any strain of *S. cerevisiae* [54–56].

The aim of this work was to identify novel genetic elements responsible for the very high acetic acid tolerance in a yeast strain previously obtained after whole-genome transformation (WGT) with gDNA from the highly acetic acid-tolerant *S. cerevisiae* strain K11 and in which *snf4*^E269*^ has been identified as the causative mutation introduced by WGT. Snf4 is a subunit of the regulatory protein kinase Snf1, which is not only known to play a central role in the glucose repression pathway but also affects many other metabolic, physiological and developmental properties in yeast [57–59]. We have performed pooled-segregant whole-genome sequence analysis after crossing the acetic acid-tolerant whole-genome transformant with another highly acetic acid-tolerant strain. Four major QTLs were mapped, in which we identified again *snf4*^E269*^, as well as interacting superior alleles of six new genes, *TRT2*, *MET4*, *IRA2* and *RTG1*, and a combination of two genes *MSH2* and *HAL9,* as causative elements by RHA.

## Results

**Fig. 1:**
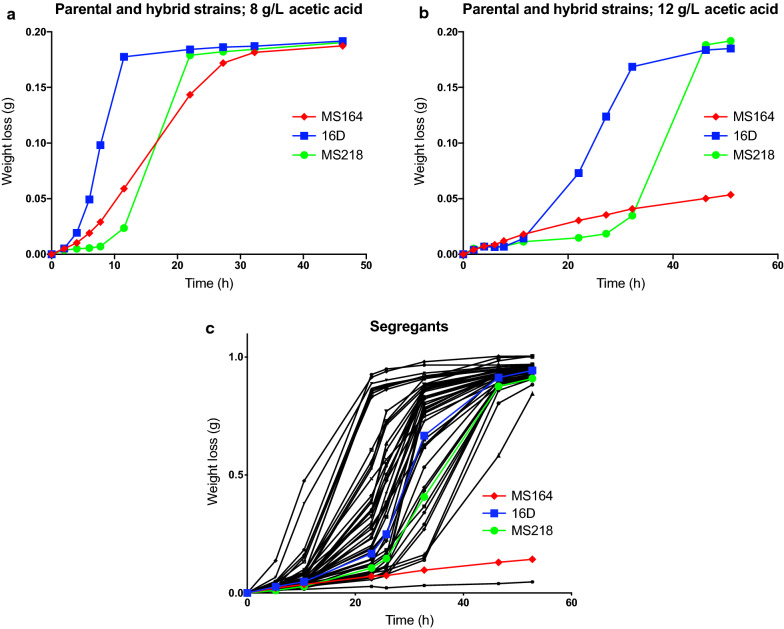
Assessment of acetic acid tolerance in small-scale fermentations with parent, diploid hybrid and segregant strains. Haploid parental strains MS164 (red,  ) and 16D (blue,  ), diploid hybrid MS218 (green,  ) and a selection of haploid segregants (black). Fermentations were performed at 35 °C, constant stirring at 120 rpm, pH 4.7 in 10 mL (**a**, **b**) or 50 mL (**c**) YPD medium with 40 g/L glucose and supplemented with 8 g/L (**a**) or 12 g/L (**b**, **c**) acetic acid

In previous work, we have identified *snf4*^E269*^ as a causative allele in strain MS164 obtained after whole-genome (WG) transformation and selection for improved acetic acid tolerance (Stojiljkovic et al., submitted for publication). We have now performed polygenic analysis with the same WG transformant MS164 crossed with the highly acetic acid-tolerant strain 16D [31] to identify novel causative alleles interacting with *snf4*^E269*^ for establishing very high acetic acid tolerance, i.e., to previously unmatched levels for *Saccharomyces cerevisiae*. The hybrid diploid strain MS218, obtained by crossing MS164 with 16D, showed intermediate acetic acid tolerance compared to that of the two parent strains (Fig. 1a, b). After sporulation of strain MS218, we selected 737 segregants and evaluated their acetic acid tolerance in 10 mL small-scale fermentations. Candidate superior segregants were then re-evaluated in 50 mL fermentations (Fig. 1c) after which 33 segregants with an acetic acid tolerance, at least as high as that of the most tolerant parent strain 16D, were selected to compose the superior pool. A random pool was composed of 200 randomly selected segregants.

The gDNA of the two pools and of the two parent strains was submitted for whole-genome sequence analysis and the reads obtained were mapped against the standard gDNA sequence of strain S288c. Subsequently, the SNPs between the two parental strains, MS164 and 16D, were identified and the variant frequency for each SNP calculated in the two pools with high precision due to the high coverage of the reads in the whole-genome sequence analysis. The SNP variant frequency in the random pool was about 50% throughout the genome.

**Fig. 2:**
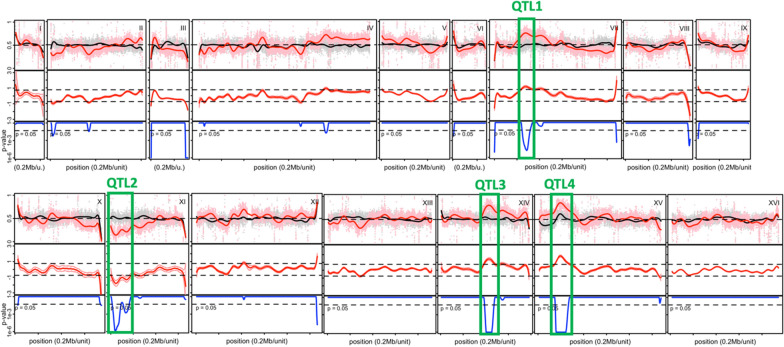
Mapping of QTLs linked to very high acetic acid tolerance. The SNP variant frequency of the superior pool (33 segregants) is shown in red, the SNP variant frequency of the random pool (200 segregants) is shown in black in the upper row. The log odds ratio with the chosen confidence interval of 0.755 is shown in red in the middle row while the calculated *p* value is shown in blue in the lower row. The four major QTLs are indicated with green rectangles. QTL1, 3 and 4 are linked to the genome of MS164 while QTL2 is linked to 16D

Subsequently, we have used the SNP variant frequency of the superior pool to map the QTLs linked to very high acetic acid tolerance for each of the 16 chromosomes (Fig. 2). This resulted in identification of four major and two minor QTLs linked to very high acetic acid tolerance. The minor QTLs were not further analyzed. Out of the four major QTLs, three were linked to the genome of MS164 and one to the genome of 16D (Fig. 2).

The four major QTLs were fine-mapped by determining the variant frequency for selected SNPs every ± 10 kb in the individual segregants using allele-specific PCR. Each of the four QTLs was then divided in blocks of genes (Table 1), which were evaluated for involvement in determining high acetic acid tolerance in fermentations by bulk-RHA. The results are indicated in Table 1 for all blocks of genes in each QTL and explained in detail with the corresponding fermentation performance (see below). In QTL1, 2 and 3 a single block was identified as causative, while in QTL4 three blocks were identified as causative. Next, we proceeded with single gene-RHA to identify the causative gene in each block.

**Table 1:** Gene blocks in the four major QTLs

QTL/link to parent	Block number	Involvement in acetic acid tolerance/link to parent	QTL/link to parent	Block number	Involvement in acetic acid tolerance/link to parent
QTL1 (Chr VII) linked to MS164	1	–	QTL3 (Chr XIV) linked to MS164	5	–
	2	–		6	–
	*3*	+/linked to MS164		7	–
	4	–	QTL4 (Chr XV) linked to MS164	*1*	+/linked to 16D
	5	–		2	–
QTL2 (Chr XI) linked to 16D	1	–		*3*	+/linked to MS164
	*2*	+/linked to 16D		4	–
	3	–		*5*	+/linked to MS164
QTL3 (Chr XIV) linked to MS164	1	–		6	–
	2	–		7	–
	3	–		8	–
	*4*	+/linked to 16D			

Overview of the 23 gene blocks within the four major QTLs and their involvement in high acetic acid tolerance. Linkage to the parent strain for each QTL and gene block is also indicated

### QTL1 on chromosome VII

**Fig. 3:**
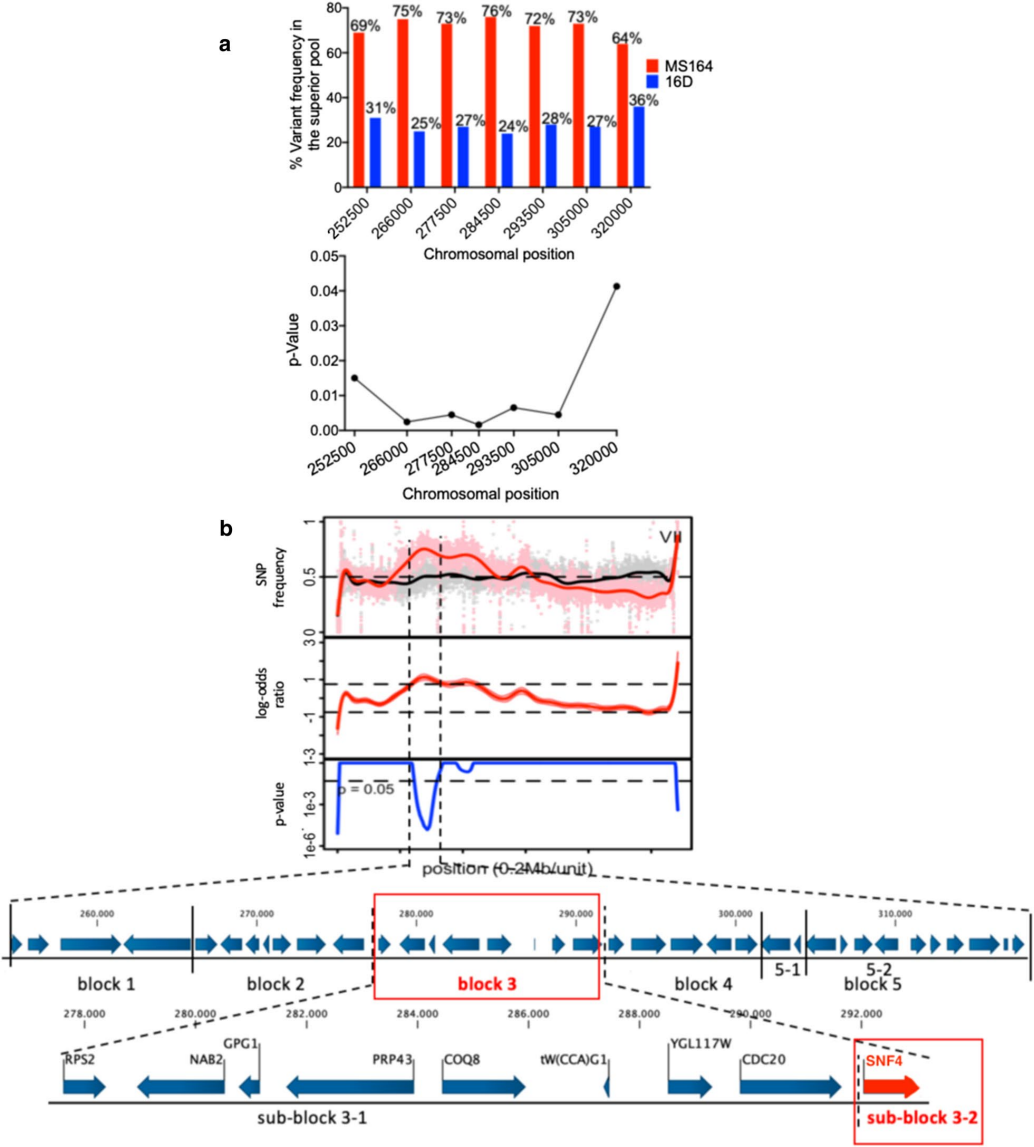
Fine mapping and linkage analysis of QTL1 on chromosome VII. **a** SNP variant frequency for selected SNPs in QTL1 and corresponding *p* values. **b** Subdivision of QTL1 into five gene blocks for construction of pairs of hemizygous hybrid strains for evaluation by bulk-RHA. A detailed view of the nine genes present in the causative block 3 is shown at the bottom. Causative blocks and genes are indicated in red

QTL1, located on chromosome VII, is about 68 kb long (from chromosomal position ± 252,000 to ± 320,000) and showed high linkage with the genome of the MS164 parent. To identify the causative gene in the QTL, we first narrowed it down by fine-mapping and calculated the corresponding *p* values, which confirmed significant linkage to the genome of MS164 (Fig. 3a). Fine-mapping (Fig. 3a) and QTL mapping (Fig. 3b) indicated that the region with the highest linkage is located at chromosomal position ± 285,000. We then divided the QTL1 in five blocks (Fig. 3b) and we constructed five pairs of hemizygous hybrid strains, each with a deletion of one of the two alleles to perform bulk-RHA. Positive transformants for block 5 could only be obtained after it was divided in two sub-blocks, 5-1 and 5-2.

**Fig. 4:**
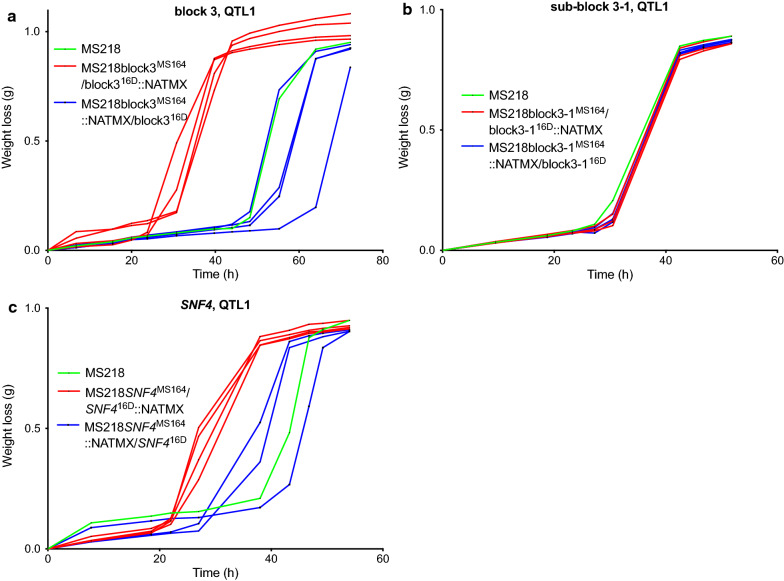
Fermentation performance in the presence of acetic acid of the causative block 3-, sub-block 3-1-, and *SNF4*-RHA strains for QTL1 on chromosome VII. **a** Causative gene block 3 and **b**, **c** gene sub-blocks 3-1 and 3-2 (*SNF4*). Hemizygous RHA strains containing the MS164 allele (red, 3 or 4 replicates), hemizygous RHA strains containing the 16D allele (blue, 3 or 4 replicates) and diploid hybrid strain MS218 (green). Fermentations were performed in 50 mL YP medium with 40 g/L glucose, supplemented with 12 g/L acetic acid, at pH 4.7, 35 °C and constant stirring at 120 rpm

We then compared the fermentation performance of the two hemizygous hybrid bulk-RHA strains for each block (Fig. 4). The fermentations were performed in YPD in the presence of a high concentration of 12 g/L acetic acid at pH 4.7 to accentuate differences in acetic acid tolerance between the strains under these relatively harsh conditions for *S. cerevisiae*. However, as has been noted previously in the literature [60], fermentations in the presence of acetic acid tend to show high variation, even between technical replicates. Hence, all replicates are shown as individual lines rather than means with standard deviations (Fig. 4). We only noticed a clear separation between the fermentation performance of the hemizygous strain with the MS164 allele (red lines) and that of the corresponding strain with the 16D allele (blue lines) for block 3 (Fig. 4c). The fermentation performance of the hybrid diploid strain (green line) was more similar to that of the hemizygous strain with the 16D allele than to that of the hemizygous strain with the MS164 allele, suggesting the presence of a recessive causative allele in strain MS164. For all the other blocks there was an overlap between the fermentation performance of the two hemizygous strains suggesting that they did not contain a causative gene (Additional file 1: Fig. S1A, B, D–F). Interestingly, block 3 overlaps with the region showing highest linkage in the fine-mapping of QTL1 (Fig. 3a).

Block 3 is almost 16 kb long, from chromosomal position ± 277,500 to ± 293,000 and consists of nine genes (Fig. 3a). Next, we divided block 3 into sub-block 3-1, containing the first eight genes and sub-block 3-2 containing only the *SNF4* gene (Fig. 3b), because we previously identified the causative mutation *snf4*^E269*^ in the *SNF4* gene in the WG transformant MS164 (Stojiljkovic et al., submitted for publication). We next evaluated the fermentation performance in the presence of a high acetic acid level of each pair of hemizygous strains for sub-blocks 3-1 and 3-2 (Fig. 4b, c). While the hemizygous strains for sub-block 3-1 did not show any difference in fermentation performance (Fig. 4b), the hemizygous strain with the *snf4*^E269*^ allele of MS164 (red lines) showed a much better fermentation performance than the corresponding strain with the *SNF4* allele of 16D (blue lines) (Fig. 4c). The fermentation performance of the hybrid diploid strain was similar to that of the hemizygous strain with the *SNF4* allele of 16D, confirming the recessive character of the *snf4*^E269*^ allele (Fig. 4c). These results show that a causative allele generated during WGT can be identified by QTL mapping and RHA analysis with mixed genetic backgrounds obtained by crossing with an unrelated strain. Snf4 is the γ subunit of the protein kinase Snf1 [59]. Mutations in *SNF4* resulted in absent utilization of glycerol, galactose and raffinose, delayed and slow utilization of sucrose, while the utilization of glucose was unaffected. Under glucose repression conditions, the *snf4* mutant did not produce detectable secreted invertase activity, while under derepression conditions, invertase activity was barely detectable [61]. Under glucose limitation and other stress conditions, Snf1 is active and phosphorylates transcription factors which regulate the expression of multiple genes linked with stress response, glucose transport and repression [62].

### QTL2 on chromosome XI

**Fig. 5:**
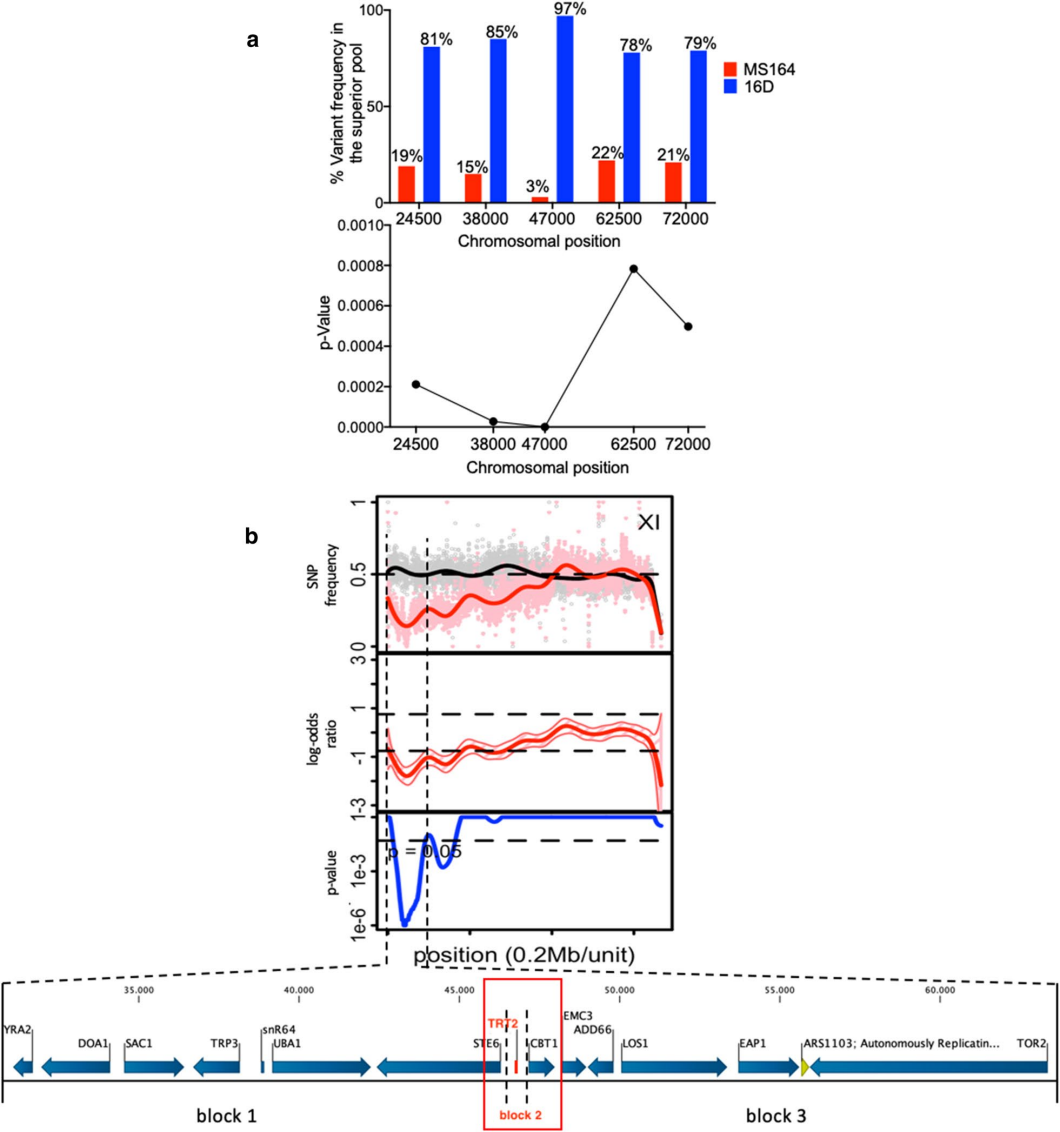
Fine mapping and linkage analysis of QTL2 on chromosome XI. **a** SNP variant frequency for selected SNPs in QTL2 and corresponding *p* values. **b** Subdivision of QTL2 into three gene blocks, of which block 2 only contains the *TRT2* gene, for construction of pairs of hemizygous hybrid strains for evaluation by bulk- or single gene-RHA. Causative block and gene are indicated in red

QTL2, located on chromosome XI, is about 48 kb long (from chromosomal position ± 24,000 to 72,000) and showed high linkage with the genome of the 16D parent. To identify the causative gene in the QTL, we first narrowed it down by fine-mapping and calculated the corresponding *p* values, which confirmed significant linkage to the genome of 16D (Fig. 5a). Fine-mapping (Fig. 5a) and QTL mapping (Fig. 5b) indicated that the region with the highest linkage is located at chromosomal position ± 47,000. We then divided the QTL2 in three blocks (Fig. 5b) and we constructed three pairs of hemizygous hybrid strains, each with a deletion of one of the two alleles to perform bulk-RHA or single gene-RHA for block 2. We chose to include only the *TRT2* gene in block 2 (± 350 bp long, between chromosomal positions ± 46,500 and ± 47,000) since a *trt2* SNP had been identified as the only causative SNP in an *S. cerevisiae* strain obtained after WGT with gDNA from *Kluyveromyces marxianus* and selection for higher thermotolerance (Deparis et al., submitted for publication). The *TRT2* gene was also located at the position with the highest linkage in the fine-mapping (Fig. 5a).

**Fig. 6:**
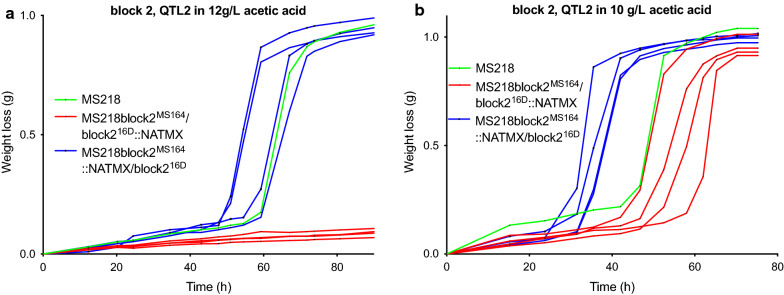
Fermentation performance in the presence of acetic acid of *TRT2*-RHA strains for QTL2 on chromosome XI. **a**, **b** Causative block 2, containing only the *TRT2* gene, in different concentrations of acetic acid. Hemizygous RHA strains containing the MS164 allele (red, 3 or 4 replicates), hemizygous RHA strains containing the 16D allele (blue, 3 or 4 replicates) and diploid hybrid strain MS218 (green). Fermentations were performed in 50 mL YP medium with 40 g/L glucose, supplemented with 12 g/L (**a**) or 10 g/L (**b**) acetic acid, at pH 4.7, 35 °C and constant stirring at 120 rpm

We next evaluated the three pairs of hemizygous hybrid strains for fermentation performance in YPD in the presence of a high concentration of acetic acid (12 g/L) at pH 4.7 (Additional file 1: Fig. S2A–C). For blocks 1 and 3, there was no significant difference in fermentation performance between the two RHA strains (Additional file 1: Fig. S2A, B). On the other hand, the hemizygous strain with the block 2/*TRT2* allele from the 16D parent showed a similar fermentation performance as the hybrid diploid strain, while the hemizygous strain with the block 2/*trt2*^*C28T*^ allele from the MS164 parent was apparently so sensitive to acetic acid that it did not even start to ferment (Fig. 6a). To avoid misinterpretation of this result, we repeated the fermentations in the presence of a lower acetic acid concentration (10 g/L). In this case, there was a clear separation between the fermentation performance of the hemizygous strain with the block 2/*TRT2* allele from the 16D parent and that of the hemizygous strain with the block 2/*trt2*^*C28T*^ allele from the MS164 parent (Fig. 6b). Hence, presence of the *trt2*^*C28T*^ allele causes a dramatic reduction in acetic acid tolerance.

### QTL3 on chromosome XIV

**Fig. 7:**
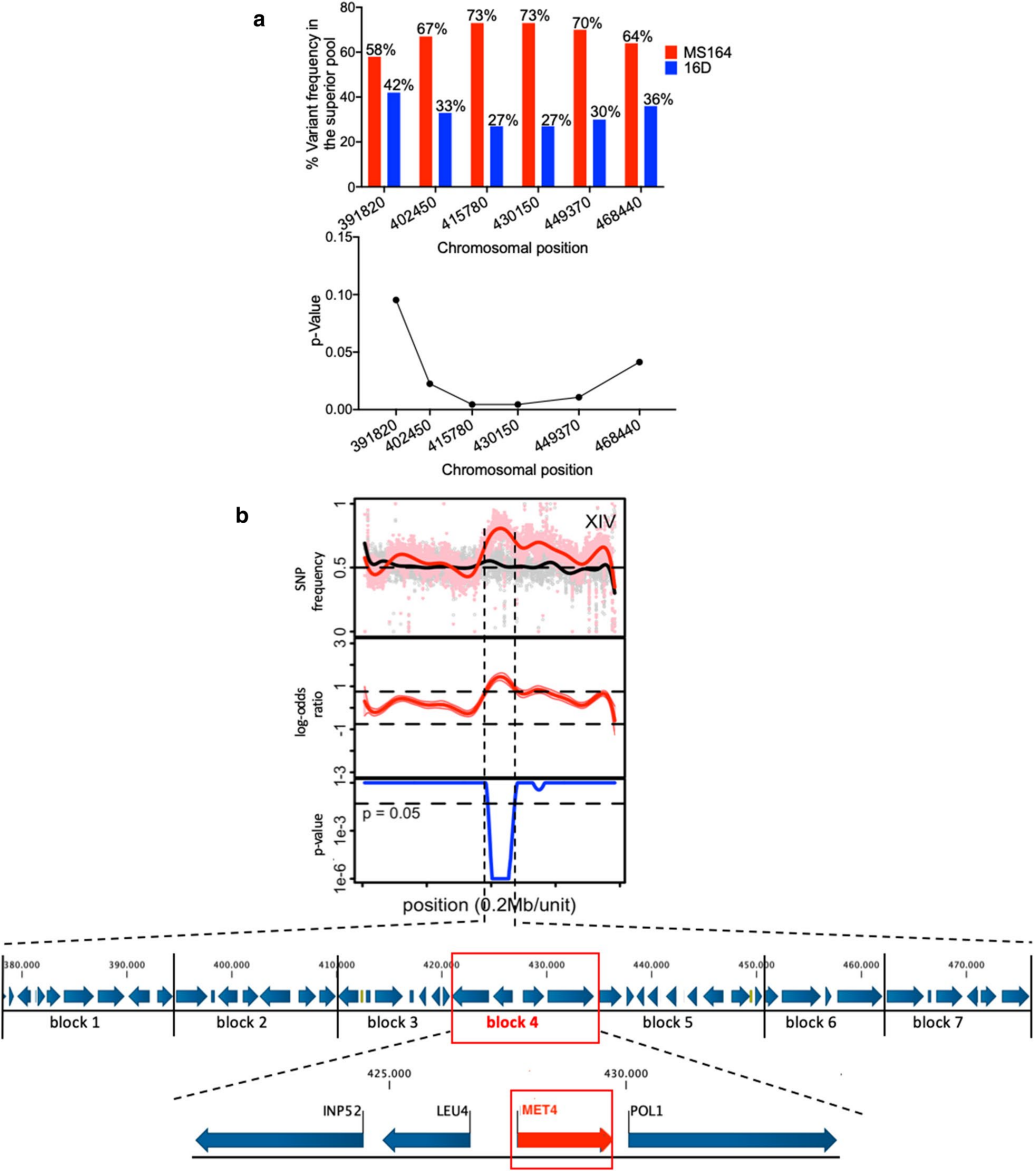
Fine mapping and linkage analysis of QTL3 on chromosome XIV. **a** SNP variant frequency for selected SNPs in QTL3 and corresponding *p* values. **b** Subdivision of QTL3 into seven gene blocks for construction of pairs of hemizygous hybrid strains for evaluation by bulk-RHA. A detailed view of the four genes present in the causative block 4 is shown at the bottom. Causative block and gene are indicated in red

QTL3, located on chromosome XIV, is about 83 kb long (chromosomal position ± 392,000 to 475,000) and showed high linkage with the genome of the MS164 parent. Using fine-mapping and determination of the corresponding *p* values, we found that the region with the highest linkage was about 20 kb long in the middle of the QTL (Fig. 7a). We divided the QTL3 in seven blocks (Fig. 7b) and constructed seven pairs of hemizygous hybrid strains, each with a deletion of one of the two alleles for each block of genes to perform bulk-RHA.

**Fig. 8:**
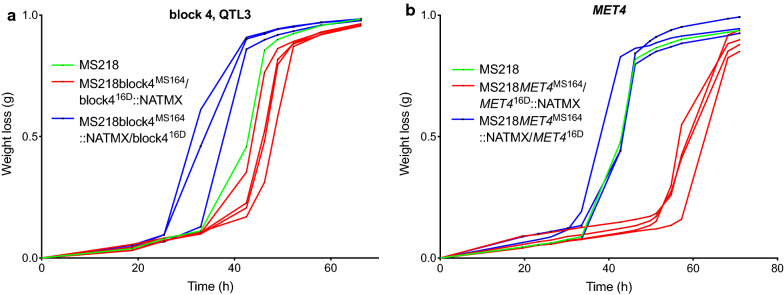
Fermentation performance in the presence of acetic acid of the causative block 4- and *MET4*-RHA strains for QTL3 on chromosome XIV. Hemizygous RHA strains containing the MS164 allele (red, 3 or 4 replicates), hemizygous RHA strains containing the 16D allele (blue, 3 or 4 replicates), and diploid hybrid strain MS218 (green). Fermentations were performed in 50 mL YP medium with 40 g/L glucose, supplemented with 12 g/L acetic acid, at pH 4.7, 35 °C and constant stirring at 120 rpm

The seven pairs of hemizygous strains were evaluated in small-scale fermentations with YPD in the presence of 1.2% acetic acid, pH 4.7 (Additional file 1: Fig. S3). The results showed that only in the case of block 4 (Fig. 8) there was a clear and consistent difference in fermentation performance between the two hemizygous strains. However, the strain with block 4 from parent 16D showed a better fermentation performance than the strain with block 4 from parent MS164, in spite of the fact that the QTL was clearly linked to the genome of parent MS164. The result for block 5 was not consistent because of the great variability for the strains with the allele from parent MS164 and because one of the three strains behaved similarly as the strains with the block from parent 16D. However, also in this case any significant link would also have been to parent 16D. This unexpected switch in linkage may be due to epistatic interference with a recessive mutation in another QTL or elsewhere in the genome, which is required for the causative allele from strain MS164 in QTL3 to have a positive effect but is complemented in the hybrid diploid RHA strain by the wild-type allele from 16D, so that the effect of the positive allele disappears and the effect of a negative allele in block 4 derived from MS164 can be manifested.

Hence, we decided to identify the superior mutant allele in block 4 derived from parent 16D. Block 4 is ± 14 kb long, from chromosomal position ± 421,000 to ± 435,000 and consists of four genes (Fig. 7b). We have assessed the four genes individually by evaluating fermentation performance in the presence of a high acetic acid level for each pair of hemizygous strains (Additional file 1:Fig. S4). Of the four genes, only the RHA strains for *MET4* showed a clear and consistent difference in fermentation performance and the strain with the allele from 16D showed the best performance, consistent with the linkage of block 4 to parent 16D. The hybrid diploid strain showed the same fermentation performance as the hemizygous strain with the *MET4* allele from 16D, indicating that the *MET4* allele from MS164 is recessive (Fig. 8).

### QTL4 on chromosome XV

**Fig. 9:**
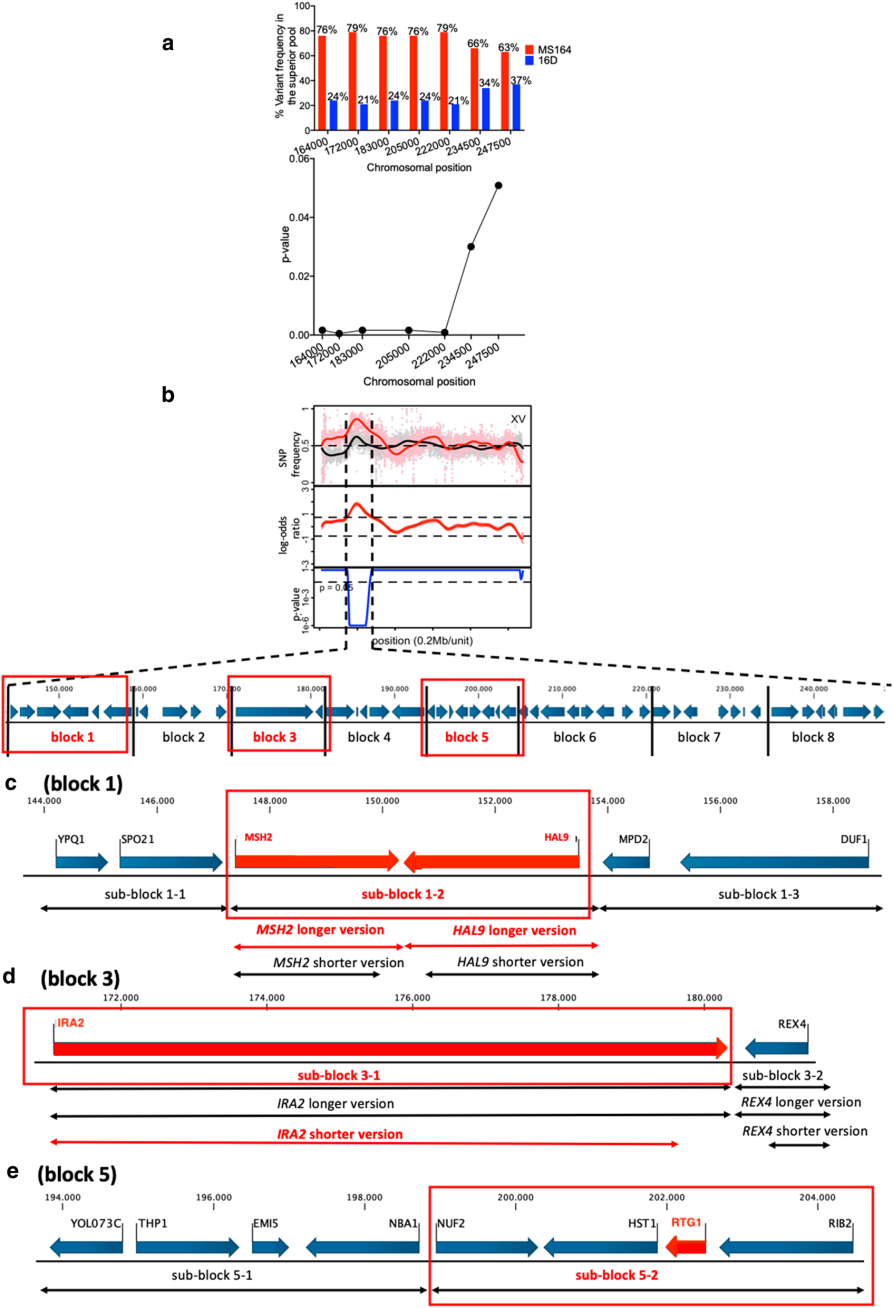
Fine mapping and linkage analysis of QTL4 on chromosome XV. **a** SNP variant frequency for selected SNPs in QTL4 and corresponding *p* values. **b** QTL4 was divided into eight gene blocks for construction of pairs of hemizygous hybrid strains for evaluation by bulk-RHA. **c**–**e** Detailed view of the genes present in the three causative blocks (and their sub-blocks) 1, 3, and 5, respectively. In gene sub-blocks 1-2 (*MSH2* and *HAL9*) (**c**), 2-1 (*IRA2*) and 2-2 (*REX4*) (**d**) each time two versions of the deletion cassettes for these genes were used: a longer version for deletion of the whole ORF and a shorter version for deletion of most of the ORF while minimizing the risk of affecting the terminator of the adjacent gene. Causative blocks and genes are indicated in red

QTL4, located on chromosome XV, is about 106 kb long (chromosomal position ± 144,000 to 250,000) and showed strong linkage with the genome of the MS164 parent. Based on the results of the fine-mapping and determination of the corresponding *p* values (Fig. 9), almost the entire region showed strong linkage to MS164. We divided the QTL4 in eight blocks and constructed eight pairs of hemizygous hybrid strains, each with a deletion of one of the two alleles to perform bulk-RHA.

**Fig. 10:**
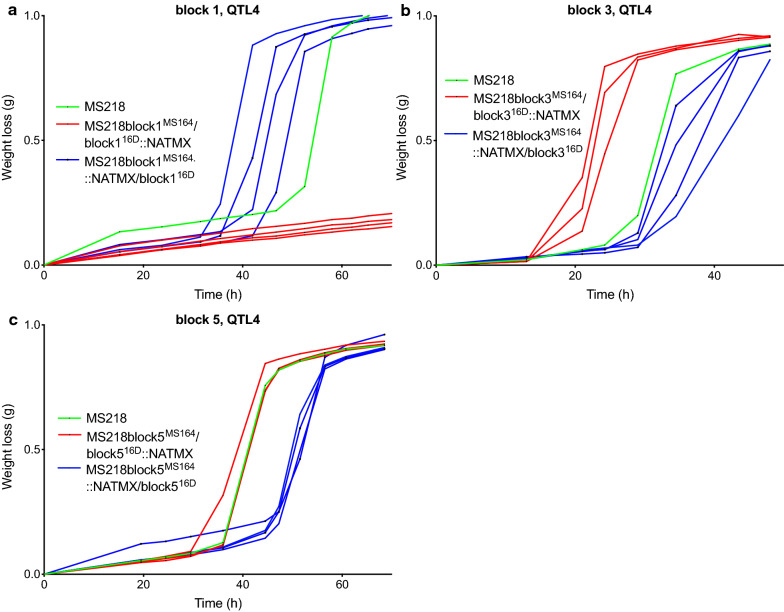
Fermentation performance in the presence of acetic acid of the causative block 1-, 3-, and 5-RHA strains for QTL4 on chromosome XV. Hemizygous RHA strains containing the MS164 allele (red, 3 or 4 replicates), hemizygous RHA strains containing the 16D allele (blue, 3 or 4 replicates) and diploid hybrid strain MS218 (green). Fermentations were performed in 50 mL YP medium with 40 g/L glucose, supplemented with 12 g/L acetic acid, at pH 4.7, 35 °C and constant stirring at 120 rpm

The eight pairs of bulk-RHA strains were evaluated for fermentation performance in YPD in the presence of 1.2% acetic acid, pH 4.7 (Additional file 1: Fig. S5). There was a clear and consistent difference in fermentation performance between the RHA strains for gene blocks 1, 3 and 5 (Fig. 10), but only gene blocks 3 and 5 were linked to the MS164 parent, as was the case for the whole QTL4, whereas gene block 1 was linked to parent 16D. Interestingly, the hybrid diploid strain showed a much better fermentation performance than the two hemizygous strains for gene block 8, suggesting that block 8 contains a gene of which a higher copy number confers higher acetic acid tolerance. There was no difference in performance, however, for the two hemizygous strains, suggesting absence of a significant difference between the alleles of the two parent strains (Additional file 1: Fig. S5H). Next, we have analyzed the three causative gene blocks in more detail to identify the causative genes.

#### Gene block 1, QTL4

**Fig. 11:**
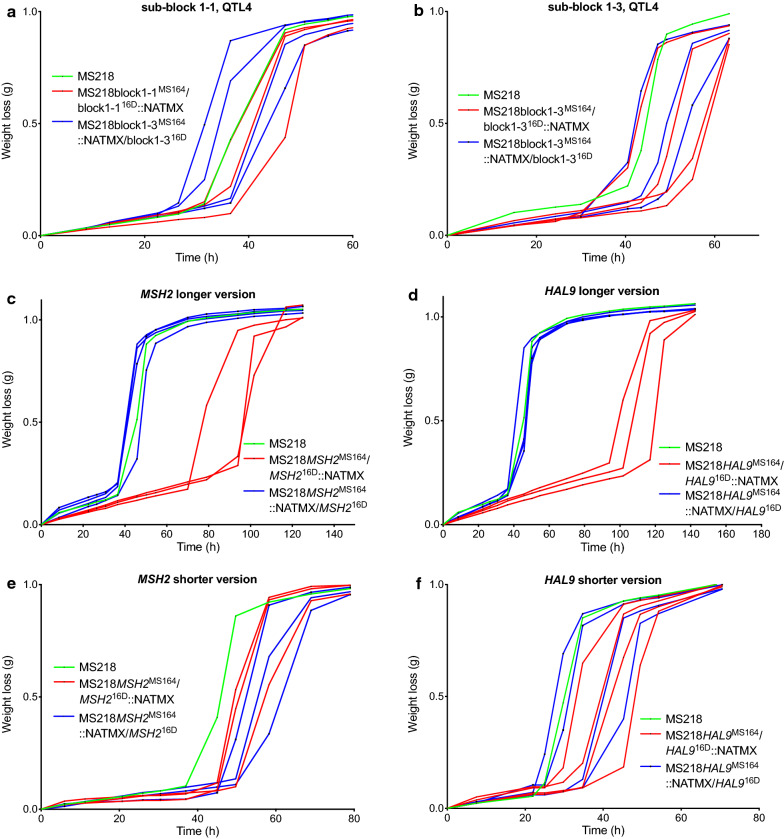
Fermentation performance in the presence of acetic acid of bulk-, *MSH2*-, and *HAL9*-RHA strains for block 1 in QTL4 on chromosome XV. **a** Sub-block 1-1, **b** sub-block 1-3 and **c**–**f** individual genes in sub-block 1-2, using in **c** a longer deletion cassette for *MSH2* and in **d** for *HAL9* and using in **e** a shorter deletion cassette for *MSH2* and in **f** for *HAL9*. Hemizygous RHA strains containing the MS164 allele (red, 3 or 4 replicates), hemizygous RHA strains containing the 16D allele (blue, 3 or 4 replicates) and diploid hybrid strain MS218 (green). Fermentations were performed in 50 mL YP medium with 40 g/L glucose, supplemented with 12 g/L acetic acid, at pH 4.7, 35 °C and constant stirring at 120 rpm

Gene block 1 in QTL4 is about 15 kb long, from the chromosomal position ± 144,000 to ± 159,000, it consists of six genes and showed high linkage with the genome of the 16D parent. We divided block 1 in three equal parts, each containing two genes (Fig. 9c) and assessed the fermentation performance of the corresponding hemizygous strains (Fig. 11). Since the hemizygous strains for sub-blocks 1-1 (Fig. 11a) and 1-3 (Fig. 11b) did not show any consistent difference in fermentation performance, we suspected that the causative gene might be located in the sub-block 1-2. Hence, we deleted the two genes, *MSH2* and *HAL9*, in this sub-block individually and evaluated the corresponding RHA strains in fermentations (Fig. 11c–f).

Because of the very short intergenic region of 121 bp between *MSH2* and *HAL9,* deletion of each ORF could have affected the terminator of the neighboring gene. To address this possible issue, we additionally made shorter deletion cassettes for most of the ORF, keeping the last part adjacent to the terminator of the neighboring gene intact (Fig. 9c). Interestingly, there was no difference in fermentation performance in the presence of acetic acid, when the two genes were deleted using the shorter deletion cassette (Fig. 11e, f). On the other hand, there was a clear difference in the fermentation performance of the two RHA strains when the complete ORFs of the *MSH2* and *HAL9* genes were individually deleted, possibly affecting proper functioning of the terminator of the neighboring gene. Hence, we can conclude that sub-block 1-2 is causative for high acetic acid tolerance and that apparently the *MSH2* and *HAL9* alleles of 16D act together in conferring high acetic acid tolerance.

#### Gene block 3, QTL4

**Fig. 12:**
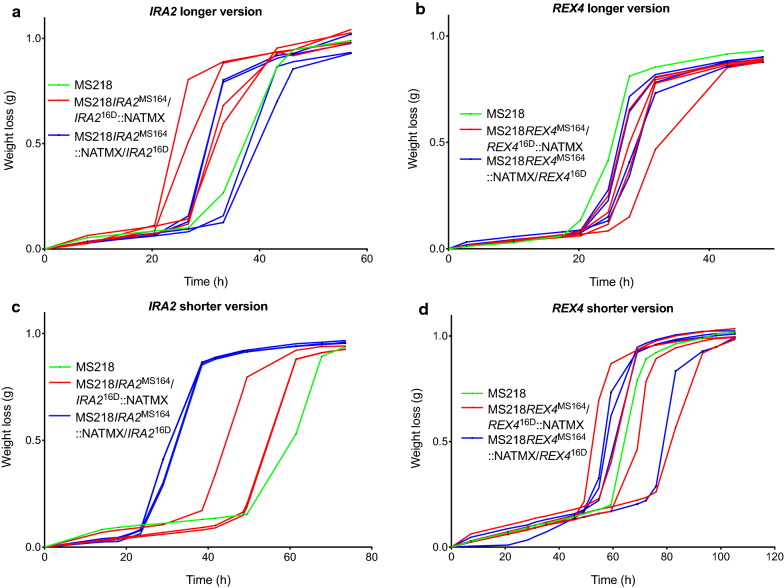
Fermentation performance in the presence of acetic acid of *IRA2*- and *REX4*-RHA strains for block 3 in QTL4 on chromosome XV. Longer (**a**, **b**) and shorter (**c**, **d**) deletion cassettes were used for *IRA2* and *REX4*. Hemizygous RHA strains containing the MS164 allele (red, 3 or 4 replicates), hemizygous RHA strains containing the 16D allele (blue, 3 or 4 replicates), and diploid hybrid strain MS218 (green). Fermentations were performed in 50 mL YP medium with 40 g/L glucose, supplemented with 12 g/L acetic acid, at pH 4.7, 35 °C and constant stirring at 120 rpm

Gene block 3 in QTL4 is about 10.5 kb long, from the chromosomal position ± 171,000 to ± 181,500; it consists of two genes, *IRA2* and *REX4*, and showed high linkage with the genome of the MS164 parent. We constructed pairs of RHA strains for *IRA2* and *REX4* individually and evaluated their fermentation performance in the presence of acetic acid (Fig. 12). As for the *MSH2* and *HAL9* genes in QTL3, we used a longer and a shorter version of the deletion cassettes for *IRA2* and *REX4*, eliminating either the complete ORF or most of the ORF to avoid interference with the terminator of the adjacent gene. In this case, deletion of the complete ORF or most of the ORF of *REX4* did not make any difference for the fermentation performance of the RHA strains and there was also no consistent difference in the performance between the RHA strains with the allele of MS164 or 16D (Fig. 12b, d). On the other hand, the RHA strains with a longer deletion of the *IRA2* gene did not show any difference in performance, whereas those with the shorter deletion showed a clear difference (Fig. 12a, c). However, the hemizygous strain with the 16D allele showed the best performance (Fig. 12c), whereas the strain with the whole gene block 3 from the MS164 parent showed the best performance (Fig. 12c). The *IRA2* allele of MS164 has an insertion of eight nucleotides at position 820 of 9248 nucleotides, creating a stop codon at position 880, and thus likely encoding a strongly truncated inactive protein. Although *IRA2* likely constitutes the causative allele in the gene block 3 in view of the frameshift mutation and the previous reports linking *IRA2* polymorphisms to differences in stress tolerance [42, 63–68], its involvement appears more complex with possible interaction with one or more neighboring genetic elements.

#### Gene block 5, QTL4

**Fig. 13:**
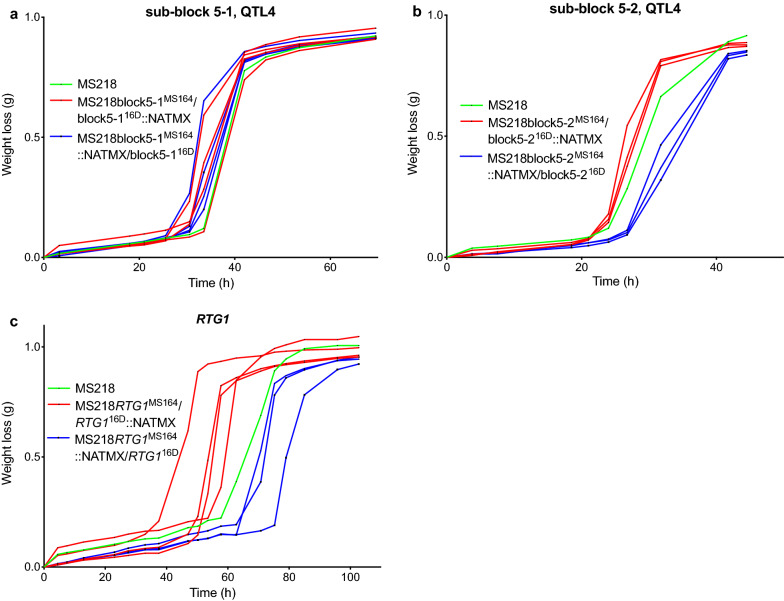
Fermentation performance in the presence of acetic acid of bulk- and *RTG1*-RHA strains for block 5 in QTL4 on chromosome XV. **a** Sub-block 5-1, **b** sub-block 5-2, and **c***RTG1* gene in sub-block 5-2. Hemizygous RHA strains containing the MS164 allele (red, 3 or 4 replicates), hemizygous RHA strains containing the 16D allele (blue, 3 or 4 replicates), and diploid hybrid strain MS218 (green). Fermentations were performed in 50 mL YP medium with 40 g/L glucose, supplemented with 12 g/L acetic acid, at pH 4.7, 35 °C and constant stirring at 120 rpm

The last causative gene block in the QTL4 (gene block 5) is about 11 kb long, from chromosomal position ± 193,500 to ± 204,500; it consists of eight genes and showed strong linkage with the genome of the MS164 parent. We divided the block in two equal parts: sub-block 5-1 and sub-block 5-2 each containing four genes (Fig. 9e). The RHA strains for the sub-block 5-1 did not show any difference in fermentation performance in the presence of acetic acid (Fig. 13a), whereas there was a clear and consistent difference in fermentation performance between the RHA strains for sub-block 5-2, with the RHA strain containing the MS164 allele showing the best performance (Fig. 13b). The latter fits with the linkage of the QTL4 and its gene block 5 to the genome of MS164. Next, we analyzed the four genes of block 5-2 individually by RHA (Additional file 1: Fig. S6). Only in case of the *RTG1* gene, a clear and consistent difference in fermentation performance between the two RHA strains was observed and the strain with the MS164 allele showed the best performance (Fig. 13c), in agreement with the linkage of the QTL, block 5 and sub-block 5-2 to the genome of MS164. The hybrid diploid strain showed an intermediate performance, suggesting that a higher *RTG1* copy number might be beneficial for high acetic acid tolerance (Fig. 13c).

### General occurrence of the SNPs in the causative alleles in other yeast strains

We have also investigated how unique or prevalent the SNPs are that are present in the causative alleles that we identified. For that purpose, we have screened the 1011 sequenced genomes of the Peter et al. [69] paper for the presence of the different SNPs. In case of *SNF4* the G to T SNP at position 805, causing the switch from E to stop, is unique. This mutation was introduced by the WGT procedure and likely represents a spontaneous mutation selected under conditions of acetic acid stress (Stojiljkovic et al., submitted for publication. The *TRT2* gene carries a G to T SNP at position 28, which apparently destabilizes the encoded tRNA^Thr^ under multiple stress conditions (this work and Deparis et al., submitted for publication). The allele of *MET4* conferring high acetic acid tolerance was derived from 16D. It contains only one SNP, which is relatively (6%) rare in other genomes: the T to C SNP at position 176. It changes a V into an A, which may be responsible for the superior character of the gene product. On the other hand, the allele of *MET4* conferring low acetic acid tolerance was derived from MS164. It contains only one SNP, which is relatively rare (10%) in other genomes: the A to G SNP at position 335. It changes a D into a G, which may be responsible for the inferior character of the gene product. The *MSH2* alleles from the two parents do not contain any non-synonymous SNPs but only 15 synonymous SNPs. Even though synonymous SNPs are generally overlooked and believed not to contribute to the phenotype, She and Jarosz [70] found that a synonymous mutation in the *UPC2* gene was responsible for panazole resistance. Unexpectedly, the authors found very similar average effect sizes of synonymous and missense mutations [70]. Therefore, the possible causative effect of synonymous SNPs present in the *MSH2* gene should not be overlooked. In addition, the *MSH2* promoter contains two SNPs/indels and the terminator contains a single SNP, which may affect the expression level of the gene. The superior allele of *HAL9* was derived from 16D and all SNPs in this allele are common to very common in other genomes. On the other hand, the inferior allele derived from MS164 has four mutations present in ≤ 7% of other genomes, of which one is entirely unique and the other one very rare (2%). Hence, it appears most likely that *HAL9* from MS164 has one or more debilitating mutations compared to the regular wild-type allele present in most strains. The inferior *IRA2* allele from MS164, conferring reduced acetic acid tolerance, contains an 8-bp insertion with a frameshift and strong truncation of the gene product as a result. In addition, it contains a very rare G to T SNP at position 289, but this has likely no relevance in the truncated gene product. Interestingly, three of the 10 strains with the rare G to T SNP also have an insertion at the same position 822, but they have a 6 bp in frame insertion rather than an 8-bp insertion causing the frameshift and early stop codon. The *RTG1* allele derived from MS164 was the superior allele but it has no conspicuous SNPs compared to the inferior allele from 16D. Both SNPs in *RTG1* are very common in other genomes. On the other hand, there are two SNPs in the promoter and one in the terminator, which may affect the expression level of the gene.

## Discussion

We have previously identified *snf4*^E269*^ as a causative allele in strain MS164 obtained after whole-genome (WG) transformation with DNA from the *S. cerevisiae* strain K11, which displays high acetic acid tolerance (Stojiljkovic et al., submitted for publication). We were surprised to identify just one single causative SNP in the transformant, that moreover was not present in the donor DNA and was thus apparently generated by spontaneous mutagenesis. The polygenic analysis performed in the present paper has identified a QTL with the *snf4*^E269*^ as causative mutation which thus confirmed its causative character. No other QTL contained a causative mutation originally present in the WG transformant. The results of the polygenic analysis thus confirm that only a single causative mutation was introduced by the WGT procedure. It is surprising that the nonsense *snf4*^E269*^ mutation, resulting in a severely truncated and thus likely inactive protein, improves acetic acid tolerance. The *SNF4* (Sucrose NonFermenting) ORF is almost 1 kb long and encodes a protein of 322 amino acids. Snf4 is an activating subunit of Snf1, which is a major component of the glucose repression pathway [71]. Hence, its inactivation will likely affect other properties of yeast such as the ability to grow on raffinose, maltose, sucrose or non-fermentable carbon sources [61, 72]. We indeed noticed that inactivation of *SNF4* caused a reduction in the growth rate on several carbon sources, while it nevertheless had a positive effect on acetic acid tolerance. Actually, screening of the yeast gene deletion collection revealed that the *snf4∆* strain was more sensitive to acetic acid [73]. The discrepancy with our results may be due to the genetic background of the strain and/or to the conditions used to evaluate acetic acid tolerance. While we have mainly used small-scale fermentations to evaluate acetic acid tolerance in view of the final goal of improving yeast acetic acid tolerance for application in industrial fermentations, the original isolation of the WG transformants was also carried out on solid medium containing different acetic acid concentrations, like the spot assays that are generally used for large-scale screening of stress tolerance characteristics in yeast. Hence, the genetic background may be a more likely cause for the difference. Mira et al. [73] screened the EUROSCARF collection of gene deletion strains in the BY4741 genetic background for acetic acid tolerance using concentrations between 0.42% and 0.66% at pH 4.5. This is much lower than the acetic acid concentration range of 0.8–1.2% at pH 4.7 that we have used in our work. Discrepancies between the effect of genetic modifications in lab strains and industrial strains have been noted previously [74]. Deletion of *SNF4* decreases the specific growth rate compared to the wild-type strain when challenged with 5% but not with 8% ethanol indicating that the dosage of the inhibitor can play an important role [75].

It was also reported that the Snf1 protein kinase is activated by acetic acid stress [73]. Besides the Snf4 subunit, other Snf1 subunits or interacting proteins may be involved to varying extents in this activation. The different genetic backgrounds may result in different levels of subunits and/or interacting proteins, and deletion of Snf4 might enhance accessibility to Snf1 of a more potent subunit or regulator. Polygenic analysis of acetic acid tolerance in a cross of *snf4∆* strains with the two genetic backgrounds could reveal the identity of protein(s) responsible for the difference in acetic acid response between the two genetic backgrounds.

Downstream targets of the Snf1–Snf4 protein kinase linked to acetic acid or stress tolerance have been identified, which may offer a possible mechanistic explanation for the enhancement of acetic acid tolerance by *snf4∆* in our genetic background. Snf1, for instance, is known to stimulate the transcription of *ADY2* [76]. Ady2 is a plasma membrane transporter involved in the uptake of the anionic form of monocarboxylic acids [77] and thus also actively transports acetate [78]. Since the deletion of *SNF1*, and potentially *SNF4*, impairs *ADY2* transcription, this could compromise active uptake of acetate resulting in lower amounts inside the cell. On the other hand, upstream regulators of *SNF4* linked to acetic acid or stress tolerance have also been identified. The *SNF4* gene, for instance, is a target of Pdr1 and Pdr3 (Additional file 1: Fig. S7), which are transcription factors that regulate the pleiotropic drug response [79] and are involved in the response to 2-methyl-4-chlorophenoxyacetic acid and 2,4-dichlorophenoxyacetic acid [80]. Also deletion of *MSN2* and *MSN4*, encoding transcription factors that activate the general stress response in *S. cerevisiae* [81], displayed downregulation of *SNF4* [82]. Further work is needed to elucidate the precise mechanism by which *SNF4* inactivation in our genetic background enhances acetic acid tolerance.

Many genes have already been identified in yeast conferring higher acetic acid tolerance upon modification or overexpression [73, 83]. However, most of these genes have been identified in laboratory strains and were involved in conferring relatively low levels of acetic acid tolerance, and often in media with a pH higher than that generally encountered in industrial fermentations, which lowers the toxicity of acetic acid. As a result, it has remained unclear what the maximal acetic acid tolerance is that can be reached in engineered industrial *S. cerevisiae* strains, especially when compared to the very high acetic acid tolerance of the food spoilage yeast *Z. bailii* [83]. Acetic acid tolerance is a highly complex trait. Polygenic analysis using parent strains with high and low acetic acid tolerance, respectively, has not only already identified multiple causative genes, but also suggested involvement of many more genes [31]. Therefore, we have performed polygenic analysis in the present work using two parent strains with high intrinsic acetic acid tolerance, to identify superior alleles that could further enhance acetic acid tolerance above the level found in the most tolerant natural *S. cerevisiae* strains in conjunction with the previously identified superior *snf4*^E269*^ allele. Polygenic analysis not only has been performed in most cases with a superior and inferior parent, but has also been successfully applied with parent strains showing similar phenotypes [53, 84, 85]. Despite using two parent strains with high intrinsic acetic acid tolerance, 33 of the 737 F1 segregants displayed even higher acetic acid tolerance than both parent strains (Fig. 1c). This transgressive segregation indicates that combining superior mutant alleles from different acetic acid-tolerant strains can lead to even more superior tolerance, which appears highly promising for development of industrial 2G strains displaying extreme acetic acid tolerance. The latter would allow application of cheaper pretreatment methodologies, which generally result in generation of higher levels of inhibitors. As expected, we indeed found QTLs and causative mutant alleles linked to both parent genomes.

Our work has now identified six additional genes linked to very high acetic acid tolerance, *TRT2*, *MET4, IRA2* and *RTG1* and a combination of *MSH2* and *HAL9*. The superior allele of *TRT2*, located in QTL2 on chromosome XI, was derived from the 16D background and is actually the wild-type allele of *TRT2*. It encodes a 72-bp essential threonine tRNA. The *trt2* allele present in strain MS164 carries a mutation that apparently destabilizes the tRNA under stress conditions, since it was also found as a causative allele in WG transformants of the MS164 strain selected for higher thermotolerance (Deparis et al., submitted for publication). Hence, in this case there appears to be a straightforward mechanistic explanation for the improvement of acetic acid tolerance by the *TRT2* gene. Moreover, it has been shown that cells modify the relative levels of tRNAs under stress conditions, suggesting that proper response and tolerance of stress requires a specific tRNA profile [86]. This may further exacerbate the drop in acetic acid tolerance caused by the *trt2* mutation.

The *MET4* gene (METhionine requiring) encodes a transcriptional activator controlling the expression of *MET3*, *MET14* and *MET16*, and is responsible for regulation of the sulfur amino acid biosynthesis pathway, e.g., for methionine [87]. *MET4* has only been linked with resistance to acetic acid in a genome-wide study of the gene deletion collection [73]. The superior *MET4* allele from 16D has one rather rare SNP (C at position 176), while the inferior allele from MS164 also has one rather rare SNP (G at position 335) (Table 2). Hence, the superior *MET4* allele from 16D may be useful to improve acetic acid tolerance in some industrial yeast strains. *MET4* is a target of Msn2 and Pdr3 (Additional file 1: Fig. S7) which makes it a potential candidate gene involved in the general stress response. It was also reported that heterozygous deletion of *MET4* caused hypersensitivity to H_2_O_2_ and decreased oxidative stress resistance [88]. In addition, the presence of auxotrophic mutations has been linked to reduced stress tolerance in yeast [89, 90].

**Table 2:** Occurrence of the SNPs identified in the causative genes in 1011 natural yeast isolates

Gene	Strain	SNP position in ORF/amino acid in gene product					
*SNF4*		593	805	807			
	16D	G/S	G/E	G/E			
	MS164	A/N	T/*	A/*			
	1011 genomes	4% G/S	100% G/E	50% G/E			
		96% A/N	0% T/*	50% A/E			
*TRT2*		28					
	16D	G					
	MS164	***T***					
	1011 genomes	100% G					
		***0% T***					
*MET4*		176	335	716	1127	1214	1244
	16D	*C/A*	A/D	T/M	T/I	G/S	A/D
	MS164	T/V	*G/G*	G/R	C/T	A/N	G/G
	1011 genomes	*6% C/A*	90% A/D	62% T/M	53% T/I	66% G/S	66% A/D
		94% T/V	*10% G/G*	38% G/R	47% C/T	34% A/N	34% G/G
*MSH2*	16D	0 non-synonymous SNPs					
	MS164						
*HAL*9		249	569	599	817	1067	1190
	16D	G/M	A/E	G/S	A/K	G/C	G/S
	MS164	A/I	G/G	A/N	***G/E***	A/Y	***A/N***
	1011 genomes	72% G/M	68% A/E	90% G/S	98% A/K	35% G/C	100% G/S
*HAL9*		28% A/I	32% G/G	10% A/N	***2% G/E***	65% A/Y	***0% A/N***
		1276	2048	2240	2315	2647	2735
	16D	G/V	G/S	T/L	C/A	T/S	A/E
	MS164	A/I	*A/N*	A/Q	T/V	*G/A*	T/V
	1011 genomes	45% G/V	94% G/S	63% T/L	16% C/A	93% T/S	69% A/E
		55% A/I	*6% A/N*	37% A/Q	84% T/V	*7% G/A*	31% T/V
		209	289	442	446	602	822-829
	16D	G/R	G/G	C/H	A/N	G/S	No insertion
*IRA2*	MS164	A/Q	***T/C***	A/N	G/S	A/N	***8*** ***bp insertion***
	1011 genomes	88% G/R	99% G/G	49% C/H	38% A/N	14% G/S	100%
		12% A/Q	***1% T/C***	51% A/N	62% G/S	86% A/N	***0%***
*RTG1*		151	334				
	16D	A/S	G/V				
	MS164	G/G	A/M				
	1011 genomes	51% A/S	67% G/V				
		49% G/G	33% A/M				

*IRA2* encodes a large protein of 3079 amino acids, which is 45% identical with the product of its paralog *IRA1* [91]. Together with Ira1, Ira2 stimulates the conversion of Ras1,2 from the GTP-active form to the GDP-bound inactive form. Ras proteins are mostly found as GDP-bound in wild-type strains and GTP-bound in *IRA* inactivation mutants. Inactivation of Ira enhances Ras activity, which increases the cAMP level and thus the activity of the cAMP-PKA pathway, which controls many properties, including thermotolerance, response to starvation, glycogen accumulation and sporulation capacity [91–93]. Spontaneous inactivating mutations in *IRA1* or *IRA2*, e.g., by insertions or deletions that cause frameshifts, have been shown previously to be responsible for variation in flocculation behavior between *S. cerevisiae* strains [65]. *IRA2* polymorphisms were also shown to be responsible for strain-dependent variation in gene expression [67], colony morphology [68], mRNA stability [64], growth and stress tolerance phenotypes [66], thermotolerance [42] and central carbon metabolites coupled with glucose uptake and ethanol production [63]. This appears similar to the frameshift mutation that we identified in *IRA2* and that affects acetic acid tolerance. The *IRA2*^*P2408T*^ allele identified by QTL analysis [42] or the *IRA2*^*YPS128*^ allele identified after experimental evolution [94] was linked to increased stress tolerance. These mutations likely enhance Ira2 GTPase-stimulating activity, leading to lower Ras activity and thus lower cAMP production and lower activity of the PKA pathway, which is well known to enhance general stress tolerance in yeast. *IRA2* is also a target of Msn2, Msn4, Pdr1 and Pdr3 (Additional file 1: Fig. S7) and therefore important in response to various stress conditions. For example, *IRA2* has been linked to heat stress [40, 95], selenite stress [96], and ethanol stress [75].

*RTG1* encodes a 177 amino acids long transcription factor (bHLH) of the ReTroGrade pathway involved in interorganelle communication between mitochondria and nucleus [97]. The RTG pathway has previously been linked to acetic acid resistance in connection with programmed cell death (PCD) induced by acetic acid stress [98]. *RTG1* was also linked to various other stresses such as selenite stress [96], heat stress [40], or ethanol stress [75].

The *MSH2* and *HAL9* genes apparently act together for their effect on acetic acid tolerance, since deletion of the individual genes did not cause any effect. On the other hand, when we disrupted both genes, affecting the terminator of the neighboring gene, we observed a clear decrease in tolerance when the alleles derived from parent MS164 were present. *MSH2* encodes a protein of 964 amino acids that binds to DNA mismatches. Together with *MSH3* and *MSH6* it initiates the mismatch repairing process [99]. Little is known about a possible connection with stress tolerance, except that the *msh2∆* strain displayed a reduced specific growth rate in the presence of 8% ethanol and osmotic challenge with 1 M NaCl [75]. The neighboring gene, *HAL9* (Halotolerance), encodes a putative transcription factor of 1030 amino acids long. Its deletion decreases salt tolerance, apparently more specifically for Li^+^, and it is required for growth in the presence of high concentrations of this cation. *HAL9* overexpression increases the expression level of *ENA1*, which encodes a pump involved in Na^+^ and Li^+^ efflux [100]. In addition, *HAL9* has been linked to heat stress [40] and selenite stress [96]. To the best of our knowledge, neither *MSH2* nor *HAL9* has previously been connected to acetic acid tolerance. However, since the RHA strain with the 16D alleles of *MSH2* and *HAL9* has the same acetic acid tolerance level as the diploid strain, while the strain with the alleles from MS164 has reduced acetic acid tolerance, the former alleles do not appear to be promising for improvement of acetic acid tolerance in other strains.

In several cases, we found a straightforward relationship between the linkage of the QTL, the causative gene block in bulk-RHA and that of the causative allele finally identified. That was true for the causative genes *SNF4* in QTL1, linked to MS164, *TRT2* in QTL2, linked to 16D, and *RTG1* in QTL4, linked to MS164. On the other hand, in QTL3 and QTL4, there was no such straightforward relationship for the other causative genes. The causative allele of *MET4*, as well as its gene block 4 in QTL3, were both linked to 16D, while the QTL was linked to MS164. The causative alleles of *MSH2* and *HAL9*, as well as their gene block 1 in QTL4, were both linked to 16D, while the QTL was linked to MS164. The causative allele of *IRA2* was linked to the 16D parent, while both the QTL4 and the gene block 3 were linked to parent MS164. These puzzling results suggest that there are epistatic interactions between the causative genes identified and other genetic elements within the QTL, which can override the effect of the causative allele on acetic acid tolerance. The underlying mechanisms remain unclear.

## Conclusions

This work has confirmed using polygenic analysis the causative nature of the *snf4*^E269*^ mutation present in a WG transformant previously selected for higher acetic acid tolerance. In addition, we have identified six other genes linked to very high acetic acid tolerance, *TRT2*, *MET4, IRA2* and *RTG1* and a combination of *MSH2* and *HAL9*, some of which have never been connected to acetic acid tolerance. Several of these genes appear to be wild-type alleles that complement defective alleles present in the other parent strain, suggesting that elimination of inferior mutant alleles might be equally important for reaching very high acetic acid tolerance as introduction of rare superior alleles. The superior alleles of *MET4* and *RTG1* might be useful for further improvement of acetic acid tolerance in specific industrial yeast strains.

## Materials and methods

### Yeast strains and cultivation media

The *Saccharomyces cerevisiae* strains used and constructed in this work are shown in Table 3. The *S. cerevisiae* strain MS164 (MAT a) was previously obtained after WGT with gDNA from the highly acetic acid-tolerant *S. cerevisiae* strain K11 and the mutant allele *snf4*^E269*^ was identified as the causative mutation introduced by WGT (Stojiljkovic et al., submitted for publication). Yeast propagation was performed in YP medium (10 g/L yeast extract, 20 g/L bacteriological peptone) with 20 g/L glucose, while fermentation was performed in YP supplemented with 40 g/L glucose. The yeast was propagated at 30 °C in an incubator with constant shaking at 200 rpm. For selection of transformants, solid YP medium was used, containing 20 g/L glucose and 15 g/L bacto agar, and supplemented with NATMX antibiotic marker. Block or single gene deletion strains were evaluated in fermentations with addition of acetic acid and with the pH corrected to 4.7, which is just below the pKa of acetic acid (4.76) to assure stringent conditions.

**Table 3:** *Saccharomyces cerevisiae* strains used and constructed in this work

Strain	Origin/background	Source
16D	Parental strain	[31]
MS164	Parental strain	Our unpublished data
MS218	Diploid hybrid	This study

Chromosome (QTL)	Genotype	Source
VII (QTL1)	MS218block1MS164/block116D::NATMX	This study
VII (QTL1)	MS218block1MS164::NATMX/block116D	This study
VII (QTL1)	MS218block2MS164/block216D::NATMX	This study
VII (QTL1)	MS218block2MS164::NATMX/block216D	This study
VII (QTL1)	MS218block3MS164/block316D::NATMX	This study
VII (QTL1)	MS218block3MS164::NATMX/block316D	This study
VII (QTL1)	MS218block4MS164/block416D::NATMX	This study
VII (QTL1)	MS218block4MS164::NATMX/block416D	This study
VII (QTL1)	MS218block5-1MS164/block5-116D::NATMX	This study
VII (QTL1)	MS218block5-1MS164::NATMX/block5-116D	This study
VII (QTL1)	MS218block5-2MS164/block5-216D::NATMX	This study
VII (QTL1)	MS218block5-2MS164::NATMX/block5-216D	This study
VII (QTL1)	MS218block3-1MS164/block3-116D::NATMX	This study
VII (QTL1)	MS218block3-1MS164::NATMX/block3-116D	This study
VII (QTL1)	MS218SNF4MS164/SNF416D::NATMX	This study
VII (QTL1)	MS218 SNF4MS164::NATMX/SNF416D	This study
XI (QTL2)	MS218block1MS164/block116D::NATMX	This study
XI (QTL2)	MS218block1MS164::NATMX/block116D	This study
XI (QTL2)	MS218block2MS164/block216D::NATMX	This study
XI (QTL2)	MS218block2MS164::NATMX/block216D	This study
XI (QTL2)	MS218block3MS164/block316D::NATMX	This study
XI (QTL2)	MS218block3MS164::NATMX/block316D	This study
XIV (QTL3)	MS218block1MS164/block116D::NATMX	This study
XIV (QTL3)	MS218block1MS164::NATMX/block116D	This study
XIV (QTL3)	MS218block2MS164/block216D::NATMX	This study
XIV (QTL3)	MS218block2MS164::NATMX/block216D	This study
XIV (QTL3)	MS218block3MS164/block316D::NATMX	This study
XIV (QTL3)	MS218block3MS164::NATMX/block316D	This study
XIV (QTL3)	MS218block4MS164/block416D::NATMX	This study
XIV (QTL3)	MS218block4MS164::NATMX/block416D	This study
XIV (QTL3)	MS218block5MS164/block516D::NATMX	This study
XIV (QTL3)	MS218block5MS164::NATMX/block516D	This study
XIV (QTL3)	MS218block6MS164/block616D::NATMX	This study
XIV (QTL3)	MS218block6MS164::NATMX/block616D	This study
XIV (QTL3)	MS218block7MS164/block716D::NATMX	This study
XIV (QTL3)	MS218block7MS164::NATMX/block716D	This study
XIV (QTL3)	MS218INP52MS164/INP5216D::NATMX	This study
XIV (QTL3)	MS218INP52MS164::NATMX/INP5216D	This study
XIV (QTL3)	MS218LEU4MS164/LEU416D::NATMX	This study
XIV (QTL3)	MS218LEU4MS164::NATMX/LEU416D	This study
XIV (QTL3)	MS218MET4MS164/MET416D::NATMX	This study
XIV (QTL3)	MS218MET4MS164::NATMX/MET416D	This study
XIV (QTL3)	MS218POL1MS164/POL116D::NATMX	This study
XIV (QTL3)	MS218POL1MS164::NATMX/POL116D	This study
XV (QTL4)	MS218block1MS164/block116D::NATMX	This study
XV (QTL4)	MS218block1MS164::NATMX/block116D	This study
XV (QTL4)	MS218block2MS164/block216D::NATMX	This study
XV (QTL4)	MS218block2MS164::NATMX/block216D	This study
XV (QTL4)	MS218block3MS164/block316D::NATMX	This study
XV (QTL4)	MS218block3MS164::NATMX/block316D	This study
XV (QTL4)	MS218block4MS164/block416D::NATMX	This study
XV (QTL4)	MS218block4MS164::NATMX/block416D	This study
XV (QTL4)	MS218block5MS164/block516D::NATMX	This study
XV (QTL4)	MS218block5MS164::NATMX/block516D	This study
XV (QTL4)	MS218block6MS164/block616D::NATMX	This study
XV (QTL4)	MS218block6MS164::NATMX/block616D	This study
XV (QTL4)	MS218block7MS164/block716D::NATMX	This study
XV (QTL4)	MS218block7MS164::NATMX/block716D	This study
XV (QTL4)	MS218block7MS164/block716D::NATMX	This study
XV (QTL4)	MS218block7MS164::NATMX/block716D	This study
XV (QTL4)	MS218block1-1MS164/block1-116D::NATMX	This study
XV (QTL4)	MS218block1-1MS164::NATMX/block1-116D	This study
XV (QTL4)	MS218block1-3MS164/block1-316D::NATMX	This study
XV (QTL4)	MS218block1-3MS164::NATMX/block1-316D	This study
XV (QTL4)	MS218MSH2MS164/MSH216D::NATMX	This study
XV (QTL4)	MS218MSH2MS164::NATMX/MSH216D	This study
XV (QTL4)	MS218HAL9MS164/HAL916D::NATMX	This study
XV (QTL4)	MS218HAL9MS164::NATMX/HAL916D	This study
XV (QTL4)	MS218IRA2MS164/IRA 216D::NATMX	This study
XV (QTL4)	MS218IRA 2MS164::NATMX/IRA 216D	This study
XV (QTL4)	MS218REX4MS164/REX416D::NATMX	This study
XV (QTL4)	MS218REX4MS164::NATMX/REX416D	This study
XV (QTL4)	MS218block5-1MS164/block5-116D::NATMX	This study
XV (QTL4)	MS218block5-1MS164::NATMX/block5-116D	This study
XV (QTL4)	MS218block5-2MS164/block5-216D::NATMX	This study
XV (QTL4)	MS218block5-2MS164::NATMX/block5-216D	This study
XV (QTL4)	MS218NUF2MS164/NUF216D::NATMX	This study
XV (QTL4)	MS218NUF2MS164::NATMX/NUF216D	This study
XV (QTL4)	MS218HST1MS164/HST116D::NATMX	This study
XV (QTL4)	MS218HST1MS164::NATMX/HST116D	This study
XV (QTL4)	MS218RTG1MS164/RTG116D::NATMX	This study
XV (QTL4)	MS218RTG1MS164::NATMX/RTG116D	This study
XV (QTL4)	MS218RIB2MS164/RIB216D::NATMX	This study
XV (QTL4)	MS218RIB2MS164::NATMX/RIB216D	This study

### Crossing, sporulation, and assembly of segregant pools

Two haploid strains 16D (MATα) [31] and MS164 (MATa), both with high tolerance to acetic acid, were mixed and incubated on a YPD plate at 30 °C. After 8 h, the zygotes were isolated using a micromanipulator and grown up for 2 days at 30 °C. The ploidy of the diploid hybrid strain MS218 obtained was confirmed by PCR. MS218 was subsequently sporulated on the agar plate with 10 g/L potassium acetate and 15 g/L bacto agar (pH 6.0) for several days at 23 °C. Sporulated cells were treated with lyticase to degrade the ascus wall. Tetrad dissection was performed using a micromanipulator and the segregants incubated at 30 °C for 2 days.

In total, 737 segregants were tested in micro-scale fermentations (10 mL) to assess tolerance to acetic acid. YP medium supplemented with 40 g/L glucose and 12 g/L acetic acid at pH 4.7 was used. Two pools of segregants were then assembled, the superior pool consisting of segregants with higher acetic acid tolerance than both parental strains (16D and MS164) and the random pool consisting of randomly picked segregants. Out of 737 segregants tested, the superior pool was made up of 33 superior segregants and the random pool of 200 randomly picked segregants.

### Pooled-segregant whole-genome sequence analysis, QTL mapping and fine-mapping

Segregants of the superior and random pools were individually grown in YP with 20 g/L glucose for 48 h in a shaking incubator at 30 °C till stationary phase. After measuring the optical density (OD) of each culture, equal amounts of the segregants were combined to make the superior and the random pool, respectively. The gDNA (genomic DNA) of both pools and the two parent strains was extracted with the MasterPure™ Yeast DNA Purification Kit from Epicentre according to the manufacturer’s instructions and submitted to the Beijing Genomics Institute (BGI, Hong Kong) for whole-genome sequence analysis. A library of 125 pair-end reads with an average insert length of 500 bp and a coverage of > 100 was generated with the Illumina HiSeq 2500 platform. All reads (from parent strains and pools) were assembled and mapped against the S288C reference genome using NGSEP. The Hidden Markov Model was used to identify regions linked to high acetic acid tolerance. Theoretical value for unlinked single-nucleotide polymorphism (SNP) variant frequency is 50%. Regions linked to high acetic acid tolerance, called quantitative trait loci (QTLs), deviate from 50% upward when linked to the genome of strain MS164 and from 50% downward when linked to the genome of strain 16D [45, 101]. *p* values were calculated based on the difference in variant frequency between the superior pool and the random pool for each genomic position. Once the broad QTL had been identified, we proceeded to fine-mapping to narrow down the causative region. Using allele-specific PCR, we checked for the presence of selected SNPs at an average distance of about 10 kb throughout the QTL in each segregant of the superior pool and determined for each SNP the percentage of variant frequency in the superior pool. Fine-mapping enabled us to reduce significantly the number of genes to be investigated in order to identify the causative gene in the QTL.

#### Targeted block or gene deletion

MS218 is a hybrid diploid strain obtained by crossing the two haploid strains 16D and MS164. For bulk or single gene-RHA, a single gene or a block of genes derived from one of the two parents was deleted. In this way, two hemizygous diploid strains carrying a single copy of either the 16D- or the MS164-derived allele or block of alleles, were created. The allele(s) were deleted using the deletion cassettes amplified from the plasmids with the NATMX antibiotic marker and containing 60 bp flanking regions for homologous recombination. The amplification was done by PCR using Q5 enzyme. An overnight culture of the diploid parent strain MS218 was transformed with a deletion cassette containing the NATMX antibiotic marker using the LiAc/SS-DNA/PEG method [102, 103]. The transformed culture was plated on solid YPD medium containing the nourseothricin antibiotic and the transformants were grown for 2 days at 30 °C. The gDNA of the transformants was isolated using the PCI (Phenol:Chloroform:Isoamyl Alcohol 25:24:1) method. The transformants were subsequently verified for correct deletion of the single gene or block of genes by two different PCRs. The first PCR used primers binding inside the antibiotic resistance marker and outside the gene or block of genes, while the second PCR used primers inside and outside the gene or block of genes, to verify that only one copy of the gene or block of genes was deleted. When both PCRs gave a positive result, we proceeded to allele-specific PCR to determine which one of the two alleles was still present.

### Allele-specific PCR and Sanger sequencing

Allele-specific PCR was used to fine-map the QTLs. The variant frequency of selected SNPs at an average distance of 10 kb throughout the QTL was determined in each segregant of the superior pool. We also performed allele-specific PCR to identify the remaining allele(s) present in bulk or single gene-RHA strains. The forward primer differed in at least one allele-specific SNP at the 3′ end, either specific for 16D or for MS164. Both primers contained an extra mismatch at the third nucleotide position from the 3′ end to increase specificity. The reverse primer was common for both PCRs and was designed to bind at a distance of about 500 bp. After evaluation of the RHA strains by allele-specific PCR and identification of at least three correct isolates of each hemizygous strain, single cells were picked using a micromanipulator, grown up and submitted for final verification to Sanger sequencing.

### Small-scale fermentations

Strains were evaluated in 10 mL (for segregant screening) or 50 mL (all other experiments) small-scale fermentations for acetic acid tolerance under semi-anaerobic conditions. The medium used was YP containing 40 g/L glucose and various concentrations of acetic acid, with initial pH adjusted to 4.7 using 4 M KOH. Yeast cells used were pre-grown in YP with 20 g/L glucose for 48 h at 30 °C till stationary phase. After measuring the OD of each culture, the correct volume needed was calculated and the cells were harvested by centrifugation at 3000 rpm for 5 min at 4 °C. Starting OD of the fermentations was 2, corresponding to approximately 0.5 g dry weight/L cell density. Fermentations were performed at 35 °C with continuous stirring at 120 rpm in either 10 mL or 50 mL volume. Fermentation performance was assessed by measuring weight loss of the culture, which corresponds to CO_2_ release. In 10 mL fermentations, the maximum weight loss was 0.2 g while in 50 mL fermentations the maximum weight loss was 1.0 g.

## Supplementary information

**Additional file 1: Figure S1.** Fermentation performance in the presence of acetic acid of bulk-RHA strains for QTL1 on chromosome VII. Hemizygous RHA strains containing the MS164 allele (red, 3 or 4 replicates), hemizygous RHA strains containing the 16D allele (blue, 3 or 4 replicates) and diploid hybrid strain MS218 (green). Fermentations were performed in 50 mL YP medium with 40 g/L glucose, supplemented with 12 g/L acetic acid, at pH 4.7, 35 °C and constant stirring at 120 rpm. **Figure S2.** Fermentation performance in the presence of acetic acid of bulk- and *TRT2*-RHA strains for QTL2 on chromosome XI. (A) Gene block 1, (B) gene block 3, and (C,D) causative block 2, containing only the *TRT2* gene, in different concentrations of acetic acid. Hemizygous RHA strains containing the MS164 allele (red, 3 or 4 replicates), hemizygous RHA strains containing the 16D allele (blue, 3 or 4 replicates) and diploid hybrid strain MS218 (green). Fermentations were performed in 50 mL YP medium with 40 g/L glucose, supplemented with 12 g/L (A-C) or 10 g/L (D) acetic acid, at pH 4.7, 35 °C and constant stirring at 120 rpm. **Figure S3.** Fermentation performance in the presence of acetic acid of bulk-RHA strains for QTL3 on chromosome XIV. Hemizygous RHA strains containing the MS164 allele (red, 3 or 4 replicates), hemizygous RHA strains containing the 16D allele (blue, 3 or 4 replicates) and diploid hybrid strain MS218 (green). Fermentations were performed in 50 mL YP medium with 40 g/L glucose, supplemented with 12 g/L acetic acid, at pH 4.7, 35 °C and constant stirring at 120 rpm. **Figure S4.** Fermentation performance in the presence of acetic acid of RHA strains for single genes in block 4 in QTL3 on chromosome XIV. Hemizygous RHA strains containing the MS164 allele (red, 3 or 4 replicates), hemizygous RHA strains containing the 16D allele (blue, 3 or 4 replicates) and diploid hybrid strain MS218 (green). Fermentations were performed in 50 mL YP medium with 40 g/L glucose, supplemented with 12 g/L acetic acid, at pH 4.7, 35 °C and constant stirring at 120 rpm. **Figure S5.** Fermentation performance in the presence of acetic acid of bulk-RHA strains for QTL4 on chromosome XV. Hemizygous RHA strains containing the MS164 allele (red, 3 or 4 replicates), hemizygous RHA strains containing the 16D allele (blue, 3 or 4 replicates) and diploid hybrid strain MS218 (green). Fermentations were performed in 50 mL YP medium with 40 g/L glucose, supplemented with 12 g/L acetic acid, at pH 4.7, 35 °C and constant stirring at 120 rpm. **Figure S6.** Fermentation performance in the presence of acetic acid of *NUF2*-, *HST1*-, *RTG1*- and *RIB2*-RHA strains for sub-block 5-2 in QTL4 on chromosome XV. Hemizygous RHA strains containing the MS164 allele (red, 3 or 4 replicates), hemizygous RHA strains containing the 16D allele (blue, 3 or 4 replicates) and diploid hybrid strain MS218 (green). Fermentations were performed in 50 mL YP medium with 40 g/L glucose, supplemented with 12 g/L acetic acid, at pH 4.7, 35 °C and constant stirring at 120 rpm. **Figure S7.** Visualization of the transcriptional regulatory network between the most important transcription factors linked to weak acid resistance and their association with the seven genes identified in this work. We checked the link between the major transcription factors Pdr1, Pdr3, Rim101, Haa1, War1, Msn2 and Msn4, and the genes we identified, *SNF4, TRT2, MET4, MSH2 HAL9, IRA2* and *RTG1*, under various stress conditions. Black solid line indicates DNA binding only; brown solid lines indicate DNA binding + expression, dashed lines indicate expression only; green dashed lines indicate positive interaction; red dashed lines show negative interaction. Transcription factors not shown in the figure, Rim101, Haa1 and War1, were not reported as having targets among our identified genes under stress conditions. Source: http://www.yeastract.com.

## Data Availability

All data have been stored on dedicated computers at KU Leuven. All data and yeast strains are freely available upon request.

## Abbreviations

WG: Whole-genome
WGT: Whole-genome transformation
QTL: Quantitative trait locus
SNP: Single-nucleotide polymorphism
RHA: Reciprocal hemizygosity analysis
gDNA: Genomic DNA
gRNA: Guide RNA
OD: Optical density
