# Supplementary material for: Polygenic analysis of very high acetic acid tolerance in the yeast Saccharomyces cerevisiae reveals a complex genetic background and several new causative alleles
Source: Biotechnol Biofuels. 2020 Jul 16;13:126. doi: 10.1186/s13068-020-01761-5 (PMC7364526; doi:10.1186/s13068-020-01761-5)
Supplement: Supplementary file 1 — Additional file 1: Figure S1. Fermentation performance in the presence of acetic acid of bulk-RHA strains for QTL1 on chromosome VII. Hemizygous RHA strains containing the MS164 allele (red, 3 or 4 replicates), hemizygous RHA strains containing the 16D allele (blue, 3 or 4 replicates) and diploid hybrid strain MS218 (green). Fermentations were performed in 50 mL YP medium with 40 g/L glucose, supplemented with 12 g/L acetic acid, at pH 4.7, 35 °C and constant stirring at 120 rpm. Figure S2. Fermentation performance in the presence of acetic acid of bulk- and TRT2-RHA strains for QTL2 on chromosome XI. (A) Gene block 1, (B) gene block 3, and (C,D) causative block 2, containing only the TRT2 gene, in different concentrations of acetic acid. Hemizygous RHA strains containing the MS164 allele (red, 3 or 4 replicates), hemizygous RHA strains containing the 16D allele (blue, 3 or 4 replicates) and diploid hybrid strain MS218 (green). Fermentations were performed in 50 mL YP medium with 40 g/L glucose, supplemented with 12 g/L (A-C) or 10 g/L (D) acetic acid, at pH 4.7, 35 °C and constant stirring at 120 rpm. Figure S3. Fermentation performance in the presence of acetic acid of bulk-RHA strains for QTL3 on chromosome XIV. Hemizygous RHA strains containing the MS164 allele (red, 3 or 4 replicates), hemizygous RHA strains containing the 16D allele (blue, 3 or 4 replicates) and diploid hybrid strain MS218 (green). Fermentations were performed in 50 mL YP medium with 40 g/L glucose, supplemented with 12 g/L acetic acid, at pH 4.7, 35 °C and constant stirring at 120 rpm. Figure S4. Fermentation performance in the presence of acetic acid of RHA strains for single genes in block 4 in QTL3 on chromosome XIV. Hemizygous RHA strains containing the MS164 allele (red, 3 or 4 replicates), hemizygous RHA strains containing the 16D allele (blue, 3 or 4 replicates) and diploid hybrid strain MS218 (green). Fermentations were performed in 50 mL YP medium with 40 g/L glucose, supplemented wi [file 13068_2020_1761_MOESM1_ESM.docx]

**Supplementary figures**

**Figure S1.** **Fermentation performance in the presence of acetic acid of bulk-RHA strains for QTL1 on chromosome VII**. Hemizygous RHA strains containing the MS164 allele (red, 3 or 4 replicates), hemizygous RHA strains containing the 16D allele (blue, 3 or 4 replicates) and diploid hybrid strain MS218 (green). Fermentations were performed in 50 mL YP medium with 40 g/L glucose, supplemented with 12 g/L acetic acid, at pH 4.7, 35°C and constant stirring at 120 rpm.

**Figure S2. Fermentation performance in the presence of acetic acid of bulk- and *TRT2*-RHA strains for QTL2 on chromosome XI.** (A) Gene block 1, (B) gene block 3, and (C,D) causative block 2, containing only the *TRT2* gene, in different concentrations of acetic acid. Hemizygous RHA strains containing the MS164 allele (red, 3 or 4 replicates), hemizygous RHA strains containing the 16D allele (blue, 3 or 4 replicates) and diploid hybrid strain MS218 (green). Fermentations were performed in 50 mL YP medium with 40 g/L glucose, supplemented with 12g/L (A-C) or 10 g/L (D) acetic acid, at pH 4.7, 35°C and constant stirring at 120 rpm.

**Figure S3.** **Fermentation performance in the presence of acetic acid of bulk-RHA strains for QTL3 on chromosome XIV**. Hemizygous RHA strains containing the MS164 allele (red, 3 or 4 replicates), hemizygous RHA strains containing the 16D allele (blue, 3 or 4 replicates) and diploid hybrid strain MS218 (green). Fermentations were performed in 50 mL YP medium with 40 g/L glucose, supplemented with 12 g/L acetic acid, at pH 4.7, 35°C and constant stirring at 120 rpm.

**Figure S4.** **Fermentation performance in the presence of acetic acid of RHA strains for single genes in block 4 in QTL3 on chromosome XIV**. Hemizygous RHA strains containing the MS164 allele (red, 3 or 4 replicates), hemizygous RHA strains containing the 16D allele (blue, 3 or 4 replicates) and diploid hybrid strain MS218 (green). Fermentations were performed in 50 mL YP medium with 40 g/L glucose, supplemented with 12 g/L acetic acid, at pH 4.7, 35°C and constant stirring at 120 rpm.

**Figure S5.** **Fermentation performance in the presence of acetic acid of bulk-RHA strains for QTL4 on chromosome XV**. Hemizygous RHA strains containing the MS164 allele (red, 3 or 4 replicates), hemizygous RHA strains containing the 16D allele (blue, 3 or 4 replicates) and diploid hybrid strain MS218 (green). Fermentations were performed in 50 mL YP medium with 40 g/L glucose, supplemented with 12 g/L acetic acid, at pH 4.7, 35°C and constant stirring at 120 rpm.

**Figure S6.** **Fermentation performance in the presence of acetic acid of *NUF2*-, *HST1*-, *RTG1*- and *RIB2*-RHA strains for sub-block 5-2 in QTL4 on chromosome XV**. Hemizygous RHA strains containing the MS164 allele (red, 3 or 4 replicates), hemizygous RHA strains containing the 16D allele (blue, 3 or 4 replicates) and diploid hybrid strain MS218 (green). Fermentations were performed in 50 mL YP medium with 40 g/L glucose, supplemented with 12 g/L acetic acid, at pH 4.7, 35°C and constant stirring at 120 rpm.


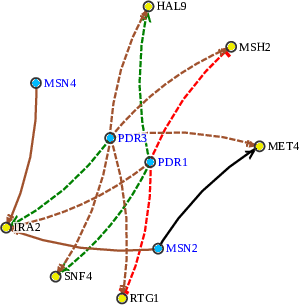


**Figure S7. Visualization of the transcriptional regulatory network between the most important transcription factors linked to weak acid resistance and their association with the seven genes identified in this work.** We checked the link between the major transcription factors Pdr1, Pdr3, Rim101, Haa1, War1, Msn2 and Msn4, and the genes we identified, *SNF4, TRT2, MET4, MSH2 HAL9, IRA2* and *RTG1*, under various stress conditions. Black solid line indicates DNA binding only; brown solid lines indicate DNA binding + expression, dashed lines indicate expression only; green dashed lines indicate positive interaction; red dashed lines show negative interaction. Transcription factors not shown in the figure, Rim101, Haa1 and War1, were not reported as having targets among our identified genes under stress conditions. Source: <http://www.yeastract.com>
